# A Web-Server of Cell Type Discrimination System

**DOI:** 10.1155/2014/459064

**Published:** 2014-01-22

**Authors:** Anyou Wang, Yan Zhong, Yanhua Wang, Qianchuan He

**Affiliations:** ^1^Lineberger Comprehensive Cancer Center, University of North Carolina, Chapel Hill, NC 27514, USA; ^2^Division of Gynecologic Oncology, Linyi Tumor Hospital, Shandong 276000, China; ^3^Department of Probability and Statistics, School of Mathematics, Beijing Institute of Technology, Beijing 100081, China; ^4^Public Health Sciences Division, Fred Hutchinson Cancer Research Center, Seattle, WA 98109, USA

## Abstract

Discriminating cell types is a daily request for stem cell biologists. However, there is not a user-friendly system available to date for public users to discriminate the common cell types, embryonic stem cells (ESCs), induced pluripotent stem cells (iPSCs), and somatic cells (SCs). Here, we develop WCTDS, a web-server of cell type discrimination system, to discriminate the three cell types and their subtypes like fetal versus adult SCs. WCTDS is developed as a top layer application of our recent publication regarding cell type discriminations, which employs DNA-methylation as biomarkers and machine learning models to discriminate cell types. Implemented by Django, Python, R, and Linux shell programming, run under Linux-Apache web server, and communicated through MySQL, WCTDS provides a friendly framework to efficiently receive the user input and to run mathematical models for analyzing data and then to present results to users. This framework is flexible and easy to be expended for other applications. Therefore, WCTDS works as a user-friendly framework to discriminate cell types and subtypes and it can also be expended to detect other cell types like cancer cells.

## 1. Instruction

Induced pluripotent stem cells (iPSCs) and embryonic stem cells (ESCs) provide important resources for medical research and applications [1]. In a stem cell research laboratory, biologists face daily the request for discriminating iPSCs, ESCs, and somatic cells (SCs). However, a user-friendly discriminant system to fill this task still remains to be further developed.

Traditional approaches like those based on single antibody (e.g., OCT4) unlikely provide a satisfactory result due to their low sensitivity and the high similarity between iPSC and ESCs [2]. Cluster analyses based on global gene expression signatures have been developed to discriminate SCs from pluripotent cells (PCs) [3–6], including iPSCs and ESCs, but this system cannot be used to discriminate iPSCs and ESCs because the gene signatures are not consistently expressed across different cell lines and conditions [5–7] and clustering is associated with the low sensitivity in determining classification [8].

Recently, we developed a quantitative discriminant system to discriminate these three cell types and their subtypes [9]. The system contains DNA-methylation biomarkers and two mathematical models, artificial neural network (ANN) and support vector machines (SVM). When appropriate biomarkers are applied, this system can discriminate SCs from PCs with 100% accuracy and can distinguish ESCs from iPSCs with an accuracy of 95%. Furthermore, this system can even accurately discriminate the subtypes of cells, such as female and male iPSCs and fetal and adult SCs [9]. Therefore, this system can be used as a framework for discriminating the three cell types and subtypes. However, running this system requires computational skills and this system could not be used directly by public users because the user input and the discrimination functions for dealing with user input have not been implemented. Here, we developed a web-server, WCTDS, to provide a user-friendly interface to allow users without any computer background to easily discriminate three cell types and their subtypes.

## 2. Materials and Methods

The WCTDS has been implemented by using Django web framework (https://www.djangoproject.com/), R 3.0 (http://www.r-project.org/), Python 2.7, and Linux shell programming, and it runs under Linux-Apache web server and mod_wsgi module. MySQL is used to build a database for communicating between user input and the data storage. Python and Django are used to implement the web interface. The whole software runs under Linux shell. The computational core, including mathematical models, data processes, and result generation, is implemented by R, which was detailed in our previous publication [9]. To speed up the computation, parallel computation was implemented to run the mathematical models.

To reduce the complexity of its usage, WCTDS runs all functions and computations behind the screen under Linux shell and only requires a simple data frame as input. The submitted data frame is directly passed by Python functions to R serial functions, including quality check, matrix preparation, parallel computation of math models, result summary, graph plots, and compressing all results in a zip file, which is sent back to web server coded by Python and Django for user download.

## 3. Results and Discussion

### 3.1. Overall View of WCTDS

**Figure 1:**
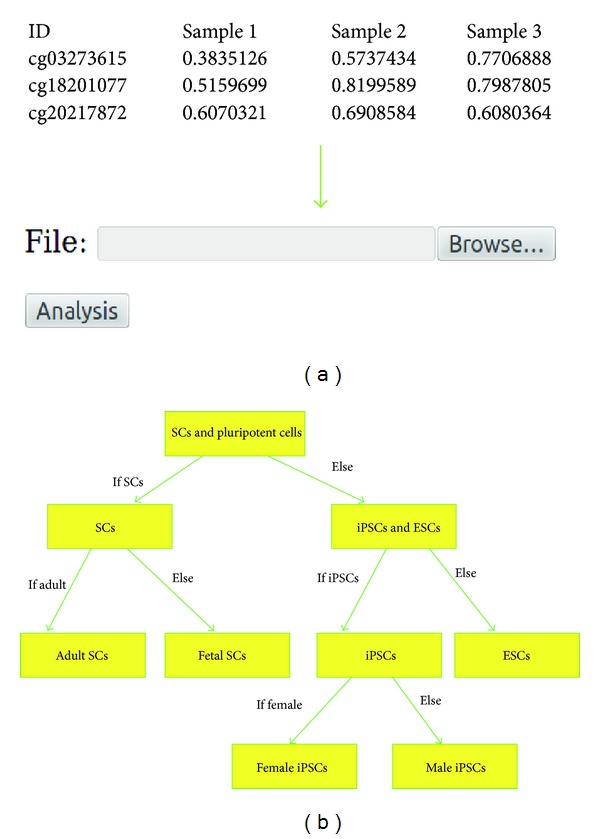
Overview of WCTDS. WCTDS provides a web interface to submit the testing data and to display an example of results and to download the entire result in a zip file. (a) After browsing the input data and clicking the analysis icon provided by the web interface, WCTDS takes care of all computational processes automatically under Linux shell behind the web interface. An example of submitted data frame format and the submission form were shown. (b) Many steps are involved in cell type discrimination. The pseudocode highlights the algorithm and discrimination processes in WCTDS.

WCTDS enables biologists without any computer backgrounds to perform cell type discriminations. WCTDS provides a user-friendly interface (Figure 1(a)) to receive a single input from users and it automatically finishes the entire discrimination processes. WCTDS runs all computational functions and multiple data processes behind the web interface under Linux shell. Typically, WCDTS first discriminates somatic cells against pluripotent stem cells (iPSCs and ESCs), then iPSCs versus ESCs, and then cell sub-type (Figure 1(b)). These steps move smoothly and efficiently because WCTDS embeds multiple computer languages, including Python, R, and Linux shell scripts (Section 2). WCTDS is also powered by Django web framework and MySQL database, which make it dynamic and flexible.

### 3.2. Features of WCTDS

The WCTDS not only includes all features that we previously published but also contains a friendly web interface for input and output as well as an implementation of a discriminant function for discriminating any user input data. Briefly, WCTDS currently at least includes the following primary features.

- A web submission form that is implemented in Python and allows a large file to submit fast.
- Submitted files are transmitted to a file that can be accepted by R codes, which is running under Linux shell that speeds up the operation.
- Convert the chromosome coordinates to the biomarker IDs.
- Two mathematical models, ANN and SVM, are run in parallel to discriminate cell types, ESCs, iPSCs, and SCs, and their subtypes including fetal and adult somatic cells and female and male stem cells.
- Random samples would be used when necessary to allow users to compare their samples against the random samples.
- The probability of discriminating samples was calculated and reported in text table and pdf figure.
- All final results including tables and figures will be compressed in a zip file and a dynamic web-link that will be provided to download the zip file if a run is successful.
- A friendly reminder when jobs are in queue and done.
- Exception handling when errors and exceptions occur for inputs.

### 3.3. Cell Type Discrimination

**Figure 2:**
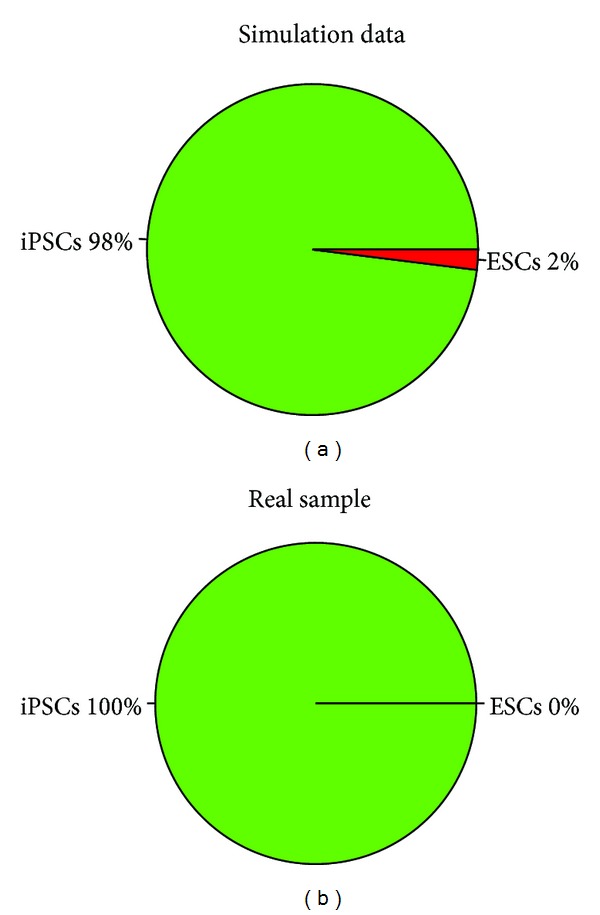
Cell type discrimination. Cell types are discriminated by estimating the probability of each type. Normally and always, the biomarkers for discriminating a pair of cell types (e.g., ESCs versus iPSCs) are used to discriminate cell types. After running the biomarkers from the start point to the end point, the probability of cell types will be summarized and a cell type will be assigned to the sample in basis of the higher probability. (a) A simulated sample with 98% probability of iPSCs and only 2% of ESCs, so the sample is discriminated as iPSCs. (b) A real iPSC sample with 100% probability, and no misclassification was (0% ESCs) found in this sample.

To improve the accuracy of discriminating a cell type or subtype, WCTDS uses an array of biomarkers to discriminate an input sample. By default, WCTDS selects 50% of total input biomarkers as a start point and uses the total input biomarkers as the end point to run the discriminant models [9]. For example, if a user inputs a data frame that contains 100 valid biomarkers for discriminating iPSCs versus ESCs, the system sorts the biomarkers and automatically selects the first 50 (50% of 100) as the start point to run the discriminant models, these discriminant processes runs until total 100 valid biomarkers are used up. After that, the probability of this sample in this group (iPSC and ESCs in this case) was calculated and cell type (iPSCs or ESCs in this case) would be assigned to the sample in basis of higher probability (Figure 2).

### 3.4. Availability and User Manual

**Figure 3:**
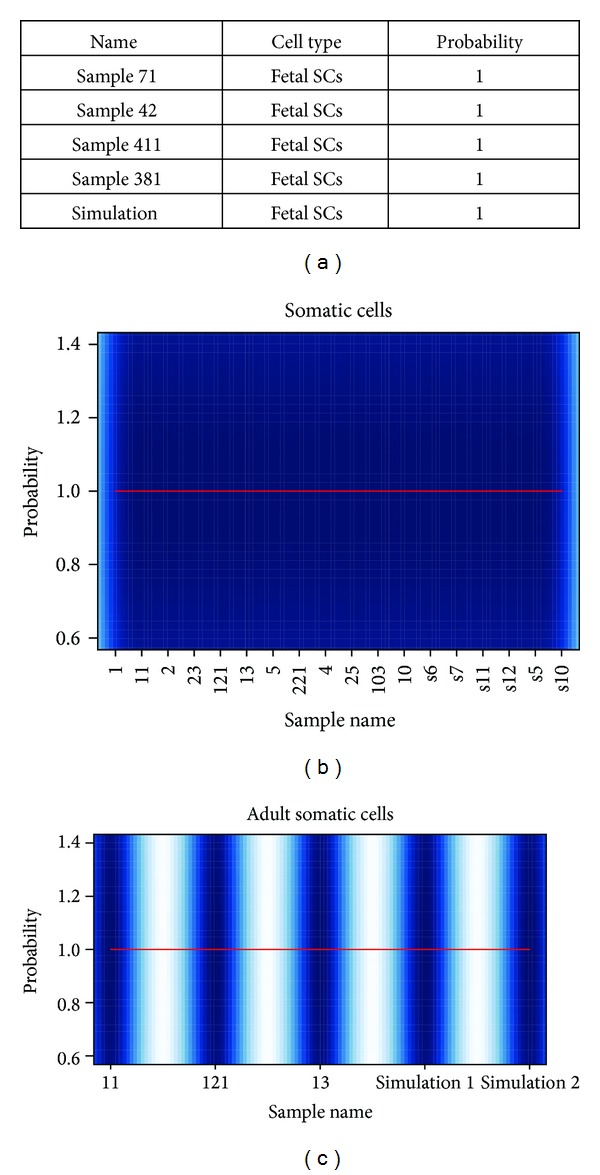
Reporting result. WCTDS reports its results in two file formats, plain text file and figure file in pdf format. (a) A partial text file shows the text file format. (b) An example of the figure format of cell type result. Color represents data distribution density. (c) An example of subtype results.

- Program name for web search engines is WCTDS.
- Project home page is  http://www.janywa.org/software/wctds/.
- Operating systems is platform independent.
- Requirements to run are any internet web-browser.
- No installation is needed.
- Running time: normally it takes around 10 minutes to run 100 samples, but, when jobs are in queue, it might take hours.
- Running WCTDS: a tab-delimited file containing user's DNA-methylation data is required to run. In the data, the first column of the data frame represents biomarker IDs, followed by one or more data columns containing sample DNA methylation data. If chromosome coordinates, instead of biomarkers IDs, are available, these chromosome coordinates can be converted into the biomarkers IDs (Figure 1(a)) [9]. The sample names must be labeled as column names (Figure 1(a)). A completed example could be found in the project home pages as shown above. In addition, some basic user info is required to fill the online form before running data. When input data is ready, users simply browse and select an input file from user's local computer and click the “upload and analysis” icon as shown online, and the program would start to run.
- Result reports: after successfully running, samples within the same cell type and sub-type will be grouped together. A summary file will be generated and reported to users in two file formats, tab-delimited text files and figure files in pdf file format for each cell type and subtype. The tab-delimited file contains sample discrimination info, including three columns, respectively, which represent sample name, predicted cell type, and estimated probability (Figure 3(a)). The pdf figure file plots the estimated probability for each sample in each cell type and subtype (Figure 3(b)).
- Result to download: all results would be packaged together into a zip file after being successfully run and a dynamic link linked to this zip file would show up for downloading if users keep their browsers open when running WCTDS. If users close their browsers, they can still download the zip package directly via http://www.janywa.org/download/yourFullFileName (e.g., myTestData.txt) after being done.

### 3.5. Flexibility and Expansion

WCTDS is implemented by Django web framework and Python and communicated by MySQL database. This implementation makes WCTDS flexible and easily expansible. WCTDS can therefore work as a base of framework to be expanded for other applications like cancer cell type discrimination. We are reusing the codes of mathematical models and are expanding this framework base to include a new system for discriminating cancers like ovarian cancer subtypes. We also welcome other scientists to use the framework to develop their applications and to add their applications into this framework in the web server.

## 4. Conclusion

We develop a web server, WCTDS, for public users to easily discriminate their cell type samples. Although the software is written in multiple computer languages including Python and R, running this system does not require any computational background. Users simply submit a tab-delimited file containing the testing data via a web submission form and the system takes care of all computational tasks and return results to be downloaded following a web link. In addition, implemented by Python-Django framework, WCTDS is flexible and expansible and it can serve as a framework to be expanded for other applications. Thereafter, this WCTDS is a user-friendly system to discriminate cell types and subtype and it can be applied to discriminate cell types in any biological experiments, not limited to stem cell researches.
